# Association of the rs738409 polymorphism in *PNPLA3 *with liver damage and the development of nonalcoholic fatty liver disease

**DOI:** 10.1186/1471-2350-11-172

**Published:** 2010-12-22

**Authors:** Kikuko Hotta, Masato Yoneda, Hideyuki Hyogo, Hidenori Ochi, Seiho Mizusawa, Takato Ueno, Kazuaki Chayama, Atsushi Nakajima, Kazuwa Nakao, Akihiro Sekine

**Affiliations:** 1 EBM Research Center, Kyoto University Graduate School of Medicine, Kyoto, Japan; 2 Division of Gastroenterology, Yokohama City University Graduate School of Medicine, Yokohama, Japan; 3 Department of Medicine and Molecular Science, Division of Frontier Medical Science, Programs for Biomedical Research, Graduate School of Biomedical Sciences, Hiroshima University, Hiroshima, Japan; 4 Research Center for Innovative Cancer Therapy, Kurume University, Kurume, Japan; 5 Department of Medicine and Clinical Science, Kyoto University Graduate School of Medicine, Kyoto, Japan

## Abstract

**Background:**

In a genome-wide association scan, the single-nucleotide polymorphism (SNP) rs738409 in the patatin-like phospholipase 3 gene (*PNPLA3*) was strongly associated with increased liver fat content. We investigated whether this SNP is associated with the occurrence and progression of nonalcoholic fatty liver disease (NAFLD) in the Japanese population.

**Methods:**

SNP rs738409 was genotyped by the Taqman assay in 253 patients with NAFLD (189 with nonalcoholic steatohepatitis [NASH] and 64 with simple steatosis) and 578 control subjects. All patients with NAFLD underwent liver biopsy. Control subjects had no metabolic disorders. For a case-control study, the *χ*^2^-test (additive model) was performed. Odds ratios (ORs) were adjusted for age, gender, and body mass index (BMI) by using multiple logistic regression analysis with genotypes (additive model), age, gender, and BMI as the independent variables. Multiple linear regression analysis was performed to test the independent effect of risk allele on clinical parameters while considering the effects of other variables (age, gender, and BMI), which were assumed to be independent of the effect of the SNP.

**Results:**

The risk allele (G-allele) frequency of rs738409 was 0.44 in the control subjects and 0.60 in patients with NAFLD; this shows a strong association with NAFLD (additive model, *P *= 9.4 × 10^-10^). The OR (95% confidence interval) adjusted for age, gender, and BMI was 1.73 (1.25-2.38). Multiple linear regression analysis indicated that the G-allele of rs738409 was significantly associated with increases in aspartate transaminase (AST) (*P *= 0.00013), alanine transaminase (ALT) (*P *= 9.1 × 10^-6^), and ferritin levels (*P *= 0.014), and the fibrosis stage (*P *= 0.011) in the patients with NAFLD, even after adjustment for age, gender, and BMI. The steatosis grade was not associated with rs738409.

**Conclusions:**

We found that in the Japanese population, individuals harboring the G-allele of rs738409 were susceptible to NAFLD, and that rs738409 was associated with plasma levels of ALT, AST, and ferritin, and the histological fibrosis stage. Our study suggests that *PNPLA3 *may be involved in the progression of fibrosis in NAFLD.

## Background

Nonalcoholic fatty liver disease (NAFLD) is now recognized as an important health concern [1,2]. NAFLD has a broad spectrum of manifestations, ranging from simple steatosis, its inflammatory counterpart nonalcoholic steatohepatitis (NASH), fibrosis/cirrhosis, to hepatocellular carcinoma. Excess fat accumulation in the liver is observed in 20-30% of the population of American and European countries where NASH is associated with approximately 1-3% of the population [3], and NAFLD is now considered to be a part of the metabolic syndrome [4]. According to recent data, fatty liver may not only be a manifestation of, but also induce insulin resistance by producing specific proteins such as, fetuin-A [5-7]. Genetic as well as environmental factors are important in the development of NAFLD [8-11]. Single-nucleotide polymorphisms (SNPs) are useful tools for identifying genetic factors and have been intensively investigated in various common diseases. Variations in peroxisome proliferator-activated receptor γ coactivator 1α (*PPARGC1A*), angiotensin II type 1 receptor (*ATGR1*), and nitric oxide synthase 2 (inducible) (*NOS2*) genes were found to be associated with NAFLD in Japanese individuals [12-14]. Variations in the adiponectin receptor 1 (*ADIPOR1*) and diacylglycerol *O*-acyltransferase 2 (*DGAT2*) genes have been reported to be associated with fatty liver and insulin resistance in the Caucasian population [15,16].

Recent genome-wide association studies revealed that the genetic variation rs738409 (I148M) in *PNPLA3 *influences NAFLD and plasma levels of liver enzymes [17-19]; replication studies suggesting the same have also been reported [20-26]. However, the association of rs738409 with the development and severity of NAFLD in the Asian population has not yet been reported. In the present study, we investigated the association between rs738409 in *PNPLA3 *and NAFLD in the Japanese population and found significant associations between rs738409 and NAFLD, plasma levels of liver enzymes, and liver fibrosis.

## Methods

### Subjects

In the present study, the subjects were 253 Japanese patients with NAFLD (189 with NASH and 64 with simple steatosis), and 578 healthy Japanese individuals (controls). All of the 253 patients with NAFLD underwent liver biopsy. Liver biopsy tissues were stained with hematoxylin and -eosin, Masson's trichrome, and reticulin silver stain. The histological criterion for the diagnosis of NAFLD is macrovesicular fatty change in hepatocytes with displacement of the nucleus toward the edge of the cell [27]. When more than 5% of the hepatocytes are affected by macrovesicular steatosis, patients are diagnosed as having either steatosis or steatohepatitis. In addition to steatosis, the criteria for the diagnosis of steatohepatitis are lobular inflammation and the presence of either ballooning cells or hepatic fibrosis containing perisinusoidal/pericellular fibrosis [28,29]. The degree of steatosis was graded as follows on the basis of the percentage of hepatocytes containing macrovesicular fat droplets: grade 0, no steatosis; grade 1, <33% hepatocytes containing macrovesicular fat droplets; grade 2, 33-66% of hepatocytes containing macrovesicular fat droplets; grade 3, >66% of hepatocytes containing macrovesicular fat droplets. The severity of the fibrosis was scored according to the method of Brunt [30]. The severity of the fibrosis was expressed on a 4-point scale, as follows: 0, none; 1, perivenular and/or perisinusoidal fibrosis in zone 3; 2, combined pericellular portal fibrosis; 3, septal/bridging fibrosis; 4, cirrhosis. Patients with the following diseases were excluded from this study: infectious hepatitis (hepatitis B and C, Epstein-Barr virus infection), autoimmune hepatitis, primary biliary cirrhosis, sclerosing cholangitis, hemochromatosis, α1-antitrypsin deficiency, Wilson's disease, drug-induced hepatitis, alcoholic hepatitis, and excessive alcohol consumption (present or past daily consumption of more than 20 g alcohol per day). No patient had any clinical evidence of hepatic decompensation, such as hepatic encephalopathy, ascites, variceal bleeding, or a serum bilirubin level greater than 2-fold the upper limit of normal. Among 253 NAFLD patients, 53 subjects had type 2 diabetes and 45 patients were under treatment: 13 patients being treated with pioglitazone, 6 with insulin, and the others with oral hypoglycemic agents other than pioglitazone. Two hundred nine patients had dyslipidemia and 81 were undergoing prescribed treatment: statines (67 patients), fibrates (10 patients), or other drugs (4 patients). One hundred fifty-three patients had hypertension and 54 patients were being treated with antihypertensive drugs: 36 patients were being treated with angiotensin receptor blockers (ARBs) and the others were being treated with other drugs such as calcium channel blockers.

All control subjects were confirmed to have normal liver function. Control subjects had no risk of fatty liver, which was determined from the following parameters: body mass index (BMI), <25 kg/m^2^; fasting plasma glucose, <110 mg/dL; serum triglycerides, <150 mg/dL, serum high-density lipoprotein (HDL) cholesterol, >40 mg/dL; systolic blood pressure, <130 mm Hg; and diastolic blood pressure, <85 mm Hg. The clinical features of the subjects are summarized in Tables 1 and 2.

**Table 1:** Clinical characteristics of the patients with NAFLD and control subjects

	NAFLD (n = 253)	Control (n = 578)	*P *value
Men/women	122/131	182/396	<0.0001*
Age (year)	51.7 ± 15.0 (38.0; 52.5; 64.0)	47.2 ± 14.8 (34.0; 49.0; 57.0)	<0.0001
BMI (kg/m^2^)	27.7 ± 5.3 (24.4; 27.3; 29.8)	21.3 ± 2.2 (19.8; 21.4; 23.1)	<0.0001
FBS (mg/dL)	117.1 ± 34.6 (99.0; 108.0; 124.0)	90.9 ± 7.7 (85.0; 90.5; 96.0)	<0.0001
HbA1c (%)	5.9 ± 1.2 (5.1; 5.5; 6.3)	5.0 ± 0.4 (4.8; 5.0; 5.3)	<0.0001
Total cholesterol (mg/dL)	212.3 ± 37.9 (183.0; 212.0; 237.0)	202.3 ± 34.4 (177.0; 202.0; 223.0)	0.0014
Triglycerides (mg/dL)	169.9 ± 97.7 (112.0; 148.0; 205.0)	75.7 ± 28.6 (53.0; 70.0; 95.0)	<0.0001
HDL cholesterol (mg/dL)	52.5 ± 17.4 (42.0; 49.0; 58.0)	67.4 ± 14.0 (57.0; 66.0; 75.8)	<0.0001
Systolic blood pressure (mm Hg)	126.7 ± 15.5 (114.0; 125.0; 138.0)	111.1 ± 10.2 (104.0; 112.0; 120.0)	<0.0001
Diastolic blood pressure (mm Hg)	77.5 ± 11.4 (70.0; 78.0; 86.0)	69.4 ± 7.5 (64.0; 70.0; 75.8)	<0.0001
AST (IU/L)	48.6 ± 29.7 (27.0; 37.0; 64.0)	19.9 ± 6.4 (15.0; 18.0; 23.0)	<0.0001
ALT (IU/L)	76.2 ± 50.7 (37.8; 61.0; 93.3)	15.6 ± 6.4 (11.0; 14.0; 19.0)	<0.0001

Data are represented as the mean ± SD (25^th^, 50^th^, and 75^th ^percentile). *P *values were obtained by comparing the quantitative phenotype between 2 groups (Mann-Whitney *U *test). *Men/women ratio was analyzed by Fisher's exact test. AST, aspartate transaminase; ALT, alanine transaminase; BMI, body mass index; FBS, fasting blood sugar; HbA1c, hemoglobin A1c; HDL, high-density lipoprotein; NAFLD, nonalcoholic fatty liver disease.

**Table 2:** Clinical characteristics of patients with NASH and simple steatosis

	NASH (n = 189)	Simple steatosis (n = 64)	*P *value
Men/women	99/90	23/41	0.030*
Age (year)	51.8 ± 15.7 (37.0; 52.5; 65.0)	51.3 ± 13.1 (44.0; 52.5; 61.0)	0.93
BMI (kg/m^2^)	28.5 ± 5.6 (25.3; 28.1; 30.4)	25.3 ± 3.5 (22.4; 24.7; 27.5)	<0.0001
FBS (mg/dL)	116.7 ± 31.5 (100.0; 108.0; 124.0)	118.0 ± 41.7 (95.8; 105.5; 122.0)	0.27
HbA1c (%)	5.9 ± 1.2 (5.1; 5.6; 6.3)	5.8 ± 1.3 (5.0; 5.3; 6.3)	0.22
Total cholesterol (mg/dL)	210.0 ± 37.3 (183.0; 210.0; 232.0)	218.0 ± 39.0 (189.5; 217.0; 249.0)	0.12
Triglycerides (mg/dL)	174.6 ± 103.9 (115.0; 149.0; 211.0)	158.2 ± 80.0 (104.0; 147.0; 195.5)	0.29
HDL cholesterol (mg/dL)	51.7 ± 18.1 (41.0; 48.0; 57.3)	54.6 ± 15.5 (44.0; 54.0; 60.5)	0.049
Systolic blood pressure (mm Hg)	127.8 ± 15.4 (118.0; 126.0; 140.0)	124.2 ± 15.7 (111.0; 124.0; 137.0)	0.13
Diastolic blood pressure (mm Hg)	77.8 ± 11.3 (70.0; 78.0; 85.0)	76.9 ± 11.9 (68.5; 76.0; 87.0)	0.58
AST (IU/L)	55.2 ± 31.3 (32.0; 45.0; 69.0)	32.5 ± 16.8 (21.0; 27.0; 36.3)	<0.0001
ALT (IU/L)	87.0 ± 53.2 (51.0; 76.0; 103.0)	49.7 ± 31.1 (30.8; 41.0; 47.3)	<0.0001
Iron (ng/mL)	111.7 ± 35.5 (91.0; 114.5; 134.0)	106.1 ± 26.1 (85.5; 100.0; 123.8)	0.087
Ferritin (ng/mL)	278.1 ± 246.5 (98.0; 204.4; 379.0)	181.2 ± 172.5 (84.0; 131.0; 175.2)	0.0036
Hyaluronic acid (ng/dL)	54.0 ± 68.5 (16.0; 32.0; 75.0)	26.7 ± 20.5 (9.0; 23.0; 36.5)	0.0048
Type IV collagen 7S (ng/dL)	4.9 ± 1.5 (3.9; 4.7; 5.5)	3.8 ± 0.7 (3.5; 3.7; 4.1)	<0.0001
Steatosis grade	1.7 ± 0.7 (1.0; 2.0; 2.0)	1.3 ± 0.5 (1.0; 1.0; 2.0)	<0.0001
Fibrosis stage	1.4 ± 0.8 (1.0; 1.0; 2.0)	0.1 ± 0.4 (0.0; 0.0; 0.0)	<0.0001

Data are represented as the mean ± SD (25^th^, 50^th^, and 75^th ^percentile). *P *values were obtained by comparing the quantitative phenotype between 2 groups (Mann-Whitney *U *test). *Men/women ratio was analyzed by Fisher's exact test. AST, aspartate transaminase; ALT, alanine transaminase; BMI, body mass index; FBS, fasting blood sugar; HbA1c, hemoglobin A1c; HDL; high-density lipoprotein; NASH, nonalcoholic steatohepatitis.

All the investigations performed in this study were conducted in accordance with the guidelines of the 1975 Declaration of Helsinki. Written informed consent was obtained from each subject, and the protocol was approved by the ethics committee of the Kyoto University, Yokohama City University, Hiroshima University, and Kurume University.

### Clinical and laboratory evaluation

The weight and height of the patients were measured with a calibrated scale after removing shoes and heavy clothing, if any. Venous blood samples were obtained from the subjects after an overnight fast (12 h) for the measurement of plasma glucose, serum aspartate transaminase (AST), alanine transaminase (ALT), hemoglobin A1c (HbA1c), total cholesterol, HDL cholesterol, triglycerides, iron, ferritin, hyaluronic acid, and type IV collagen 7S. All laboratory biochemical parameters were measured in a conventional automated analyzer.

### DNA preparation and SNP genotyping

Genomic DNA was extracted using Genomix (Talent Srl, Trieste, Italy) from blood samples collected from each subject. A predesigned TaqMan probe (Applied Biosystems, Foster City, CA) was purchased for genotyping of rs738409 (C_7241_10) and genotyping was carried out according to the manufacturer's protocol. The success rates of these assays were >99.0%.

### Statistical analysis

For each case-control study, the *χ*^2^-test (additive model) was performed according to Sladek *et al*. [31]. We coded genotypes as 0, 1, and 2, depending on the number of copies of the risk allele. Odds ratios (ORs) adjusted for age, gender, and BMI were calculated using multiple logistic regression analysis with genotypes, age, gender, and BMI as the independent variables. The Hardy-Weinberg equilibrium was assessed using the *χ*^2^-test [32]. Simple comparison of the clinical data between case and control groups was carried out using the Mann-Whitney *U *test. Simple comparison of the clinical data in the different genotypes was carried out using the Kruskal-Wallis test. Multiple linear regression analysis was performed to test the independent effect of risk allele on clinical parameters considering the effects of other variables (age, gender, and BMI), which were assumed to be independent of the effect of the SNP. The significance of the association between an independent and dependent variables was tested using the *t *test. The values of triglycerides, ferritin, and hyaluronic acid were logarithmically transformed before performing the multiple linear regression analysis. Statistical analysis was performed using the software R http://www.r-project.org/.

## Results

### Case-control association study

The clinical data of patients in the NAFLD and control groups are presented in Table 1. The clinical and biochemical characteristics of the patients with NASH and of those with simple steatosis are shown in Table 2. The risk allele (G-allele) frequency of rs738409 in the control subjects was 0.44, which was almost the same as that reported in the HapMap database but higher than that in other populations (such as European and African American). The risk allele frequency in the NAFLD patients was 0.60, and rs738409 showed a strong association with NAFLD (*P *= 9.4 × 10^-10^; Table 3). The OR for the heterozygotes was 1.46 and that for the homozygotes was 3.63, indicating an allele-dependent dose effect. The number of women, and the age and BMI of patients in the NAFLD group were higher than those of individuals in the control group. Even after the parameters of gender and age were adjusted for, rs738409 showed strong association with NAFLD (*P *= 1.9 × 10^-8^). When the case-control association study was performed separately in men and women, significant associations were observed (*P *= 0.00017 in men and 1.6 × 10^-6 ^in women; Additional file 1 Table S1). When the parameters of gender, age, and BMI were adjusted for, this association was significant (*P *= 0.00087). The OR (95% confidence interval [CI]) adjusted for age, gender, and BMI was 1.73 (1.25-2.38). We divided the NAFLD group into the NASH and simple steatosis groups and performed case-control association studies. The risk allele frequency in the simple steatosis group was 0.50, and that in the NASH group was 0.64, which was the highest among the 3 groups. A significant association was observed between NASH and control groups (*P *= 1.8 × 10^-11^), and between NASH and simple steatosis groups (*P *= 0.0083). Significant association with NASH was observed even after adjusting for parameters of gender and age (*P *= 5.7 × 10^-10^), and gender, age, and BMI (*P *= 0.00082). No significant association between the simple steatosis group and the control group was observed. The genotype of rs738409 was in Hardy-Weinberg equilibrium in the 3 groups (*P *> 0.05). Thus, the rs738409 genotype may be associated with the progression of NAFLD.

**Table 3:** Genotype frequencies and association tests of rs738409 in PNPLA3 in patients with NAFLD and control subjects

	Genotype CC/CG/GG	Risk allele (G) frequency	*P *value (additive model)			OR (95% CI)			HWE
				
			Unadjusted	Adjusted for age and gender	Adjusted for age, gender and BMI	Heterogyotes	Homogyotes	Additive model *	*P *value
NAFLD vs. control									
NAFLD	45/111/97	0.60	9.4 × 10^-10^	1.9 × 10^-8^	0.00087	1.46 (0.98 - 2.16)	3.63 (2.36 - 5.57)	1.73 (1.25 - 2.38)	0.18
Control	175/296/104	0.44							0.28

NASH vs. control									
NASH	26/85/78	0.64	1.8 × 10^-11^	5.7 × 10^-10^	0.00082	1.93 (1.20 - 3.12)	5.05 (3.04 - 8.37)	2.00 (1.33 - 3.00)	0.71
Control	175/296/104	0.44							0.28

NASH vs. simple steatosis									
NASH	26/85/78	0.64	0.0083	0.010	0.021	2.39 (1.14 - 4.99)	3.00 (1.38 - 6.51)	1.65 (1.08 - 2.51)	0.71
Simple steatosis	19/26/19	0.50							0.13

Simple steatosis vs. control									
Simple steatosis	19/26/19	0.50	0.18	0.24	0.16	0.81 (0.44 - 1.50)	1.68 (0.85 - 3.32)	1.37 (0.88 - 2.14)	0.13
Control	175/296/104	0.44							0.28

CI, confidence interval; NAFLD, nonalcoholic fatty liver disease; NASH, nonalcoholic steatohepatitis; HWE, Hardy-Weinberg equilibrium; OR, odds ratio. * The OR was adjusted simultaneously for age, gender, and BMI using the additive model.

### Analysis of various quantitative phenotypes with the rs738409 SNP

To investigate whether the genotypes of rs738409 were associated with clinical parameters, we compared age, BMI, fasting plasma glucose, HbA1c, total cholesterol, triglycerides, HDL cholesterol, AST, ALT, iron, ferritin, type IV collagen 7S domain, and hyaluronic acid between different genotypes in the NAFLD and control subjects. Fasting plasma glucose and serum triglycerides levels were low in patients with NAFLD harboring the risk allele (G-allele) (Table 4 Additional file 2 Table S2). In contrast, AST (*P *= 0.00017) and ALT (4.7 × 10^-5^) levels were significantly higher in these patients. Serum AST and ALT levels were higher in the control subjects harboring the risk allele, although differences were not significant. Among the serum markers of liver fibrosis, serum ferritin (*P *= 0.038) and hyaluronic acid (*P *= 0.022) were significantly higher in patients with NAFLD harboring the G-allele.

**Table 4:** Comparison of various quantitative phenotypes among the different genotypes at rs738409 in PNPLA3 in patients with NAFLD and control subjects

Quantitative phenotype		NAFLD *				Control *		
	
	CC (n = 45)	CG (n = 111)	GG (n = 97)	*P *value^§^	CC (n = 175)	CG (n = 296)	GG (n = 104)	*P *value^§^
Age (year)	53.4 ± 11.2	48.6 ± 15.0	54.5 ± 16.0	0.019	45.6 ± 14.9	47.6 ± 14.5	48.9 ± 15.7	0.17
BMI (kg/m^2^)	26.8 ± 4.9	28.0 ± 4.4	27.8 ± 6.3	0.16	21.2 ± 2.2	21.4 ± 2.1	21.4 ± 2.0	0.75
FBS (mg/dL)	121.2 ± 28.7	117.5 ± 38.2	114.5 ± 33.1	0.014	90.0 ± 7.8	91.4 ± 7.6	90.7 ± 7.4	0.23
HbA1c (%)	6.0 ± 1.2	5.9 ± 1.3	5.8 ± 1.1	0.36	5.0 ± 0.3	5.0 ± 0.4	5.1 ± 0.3	0.78
Total cholesterol (mg/dL)	217.4 ± 32.8	209.7 ± 40.6	212.6 ± 37.2	0.38	198.6 ± 34.0	203.7 ± 33.7	203.6 ± 36.6	0.25
Triglycerides (mg/dL)	175.3 ± 81.4	188.0 ± 119.5	146.2 ± 69.0	0.0055	75.7 ± 29.1	76.5 ± 29.0	72.9 ± 27.0	0.56
HDL cholesterol (mg/dL)	54.6 ± 21.0	50.6 ± 17.6	53.7 ± 15.0	0.20	65.4 ± 13.3	68.0 ± 13.9	69.1 ± 14.9	0.073
SBP (mm Hg)	128.3 ± 14.8	127.0 ± 16.0	125.6 ± 15.5	0.61	109.6 ± 10.6	112.1 ± 10.0	110.3 ± 9.7	0.033
DBP (mm Hg)	78.9 ± 11.4	77.1 ± 12.4	77.3 ± 10.2	0.88	68.6 ± 7.9	70.0 ± 7.4	69.2 ± 7.2	0.16
AST (IU/L)	34.0 ± 14.6	47.5 ± 26.4	57.5 ± 35.5	0.00017	19.3 ± 6.5	19.6 ± 6.5	21.2 ± 5.8	0.047
ALT (IU/L)	48.3 ± 24.6	76.9 ± 48.9	89.6 ± 57.0	4.7 × 10^-5^	15.1 ± 5.5	15.5 ± 6.9	16.4 ± 6.2	0.36
Iron (ng/mL)	112.4 ± 28.7	106.1 ± 34.7	113.9 ± 33.1	0.66	--	--	--	--
Ferritin (ng/mL)	168.8 ± 124.4	271.2 ± 268.6	276.3 ± 224.7	0.038	--	--	--	--
Hyaluronic acid (ng/dL)	48.4 ± 47.4	39.7 ± 55.3	57.3 ± 74.0	0.022	--	--	--	--
Type IV collagen 7S (ng/dL)	4.6 ± 1.9	4.6 ± 1.4	4.6 ± 1.2	0.76	--	--	--	--
Steatosis grade	1.5 ± 0.6	1.6 ± 0.7	1.6 ± 0.6	0.57	--	--	--	--
Fibrosis stage	0.7 ± 0.8	1.1 ± 0.9	1.2 ± 1.0	0.013	--	--	--	--

Data are represented as the mean ± SD. AST, aspartate transaminase; ALT, alanine transaminase; BMI, body mass index; DBP, diastolic blood pressure; FBS, fasting blood sugar; HbA1c, hemoglobin A1c; HDL, high-density lipoprotein; NAFLD, nonalcoholic fatty liver disease; SBP, systolic blood pressure. * The data of each quantitative phenotype from NAFLD and control subjects were compared for the different rs738409 genotypes in patients with NAFLD and control subjects. ^§^*P *values were analyzed using the Kruskal-Wallis test in each group of NAFLD and control subjects.

To investigate the effects of age, gender, and BMI, we attempted to perform linear multiple regression analysis with plasma glucose, triglycerides, AST, ALT, ferritin, and hyaluronic acid as the dependent variables. We coded genotypes as 0, 1, or 2, depending on the number of copies of the risk allele, and used age, gender, BMI, or SNP as an explanatory variable. The values of triglycerides, ferritin, and hyaluronic acid were logarithmically transformed before performing multiple linear regression analysis, since these values had skewed distribution. The G-allele of rs738409 was significantly associated with increases in AST (*P *= 0.00013) and ALT (*P *= 9.1 × 10^-6^) levels even after age, gender, and BMI were included in the model in the NAFLD group (Table 5). In the control group, the G-allele of rs738409 was not significantly associated with differences in AST and ALT levels. rs738409 was also associated with increased serum ferritin levels (*P *= 0.014) but not with hyaluronic acid levels. The G-allele was significantly associated with decreased levels of triglycerides (*P *= 0.0034). The G-allele was not associated with fasting plasma glucose after being adjusted for age and gender and/or BMI. The genotype of rs738409 may be related to the severity of NAFLD.

**Table 5:** Multiple linear regression analysis for AST, ALT, triglycerides, ferritin, hyaluronic acid, steatosis grade, and fibrosis stage

Explanatory variable	Subjects	Independent variable	β	SE	*P *value
AST	NAFLD	Genotype (additive model, 0/1/2)	10.165	2.606	0.00013
		Age (year)	-0.162	0.153	0.29
		Gender (Man/Women, 1/0)	-4.967	4.335	0.25
		BMI (kg/m^2^)	0.711	0.380	0.063
	
	Control	Genotype (additive model, 0/1/2)	0.567	0.466	0.23
		Age (year)	0.176	0.022	4.2 × 10^-14^
		Gender (Man/Women, 1/0)	0.226	0.775	0.77
		BMI (kg/m^2^)	0.120	0.167	0.47

ALT	NAFLD	Genotype (additive model, 0/1/2)	18.977	4.171	9.1 × 10^-6^
		Age (year)	-1.125	0.244	7.1 × 10^-6^
		Gender (Man/Women, 1/0)	7.571	6.938	0.28
		BMI (kg/m^2^)	0.991	0.608	0.10
	
	Control	Genotype (additive model, 0/1/2)	0.417	0.477	0.38
		Age (year)	0.120	0.023	2.5 × 10^-7^
		Gender (Man/Women, 1/0)	2.131	0.793	0.0076
		BMI (kg/m^2^)	0.370	0.171	0.031

Triglycerides	NAFLD	Genotype (additive model, 0/1/2)	-0.050	0.017	0.0035
		Age (year)	-0.003	0.001	0.0051
		Gender (Man/Women, 1/0)	0.102	0.023	0.00038
		BMI (kg/m^2^)	-0.001	0.002	0.66

Ferritin	NAFLD	Genotype (additive model, 0/1/2)	0.107	0.043	0.014
		Age (year)	0.005	0.002	0.056
		Gender (Man/Women, 1/0)	0.298	0.069	2.9 × 10^-5^
		BMI (kg/m^2^)	0.009	0.006	0.11

Hyaluronic acid	NAFLD	Genotype (additive model, 0/1/2)	0.023	0.036	0.52
		Age (year)	0.016	0.002	1.6 × 10^-12^
		Gender (Man/Women, 1/0)	-0.010	0.059	0.87
		BMI (kg/m^2^)	0.014	0.005	0.0047

Steatosis grade	NAFLD	Genotype (additive model, 0/1/2)	0.041	0.059	0.48
		Age (year)	-0.010	0.003	0.0037
		Gender (Man/Women, 1/0)	-0.106	0.098	0.28
		BMI (kg/m^2^)	0.022	0.009	0.010

Fibrosis stage	NAFLD	Genotype (additive model, 0/1/2)	0.211	0.082	0.011
		Age (year)	0.013	0.005	0.0058
		Gender (Man/Women, 1/0)	0.184	0.136	0.18
		BMI (kg/m^2^)	0.046	0.012	0.00016

AST, aspartate transaminase; ALT, alanine transaminase; BMI, body mass index; NAFLD, nonalcoholic fatty liver disease. The values of triglycerides, ferritin, and hyaluronic acid were logarithmically transformed.

### Association between rs738409 genotypes and the histological steatosis grade or fibrosis stage

We investigated the association between rs738409 genotypes and the histological steatosis grade or fibrosis stage. The results of the analysis revealed an additive increase in the fibrosis stage in the patients with the G-allele of rs738409 (*P *= 0.013 in the Kruskal-Wallis test) (Table 4 and Additional file 2 Table S2). Multiple linear regression analysis revealed that the rs738409 genotype was significantly associated with the fibrosis stage, even after age, gender, and BMI were included in this model (*P *= 0.011; Table 5). Although rs738409 was identified to be the variant associated with liver fat content, the steatosis grade was not associated with the rs738409 genotype (Table 4 Additional file 2 Table S2).

## Discussion

In 2 recent genome-wide association studies, *PNPLA3 *was found to be associated with liver-related conditions [17,18]. Association of the G-allele of rs738409 with increased liver fat content [20,22,23] and ALT levels [21,24-26] has been replicated; hence, the influence of this SNP to NAFLD was strongly suggested. The association of this SNP with the development of NAFLD and liver damage in NAFLD has been shown in the Argentinian and Caucasian populations [21,25]. There is no report on the association between rs738409 and NAFLD in the Japanese population. The results of the present study indicated that the G-allele of rs738709 is strongly associated with NAFLD (especially NASH); increased plasma levels of ALT and AST; decreased plasma levels of triglycerides; and the higher fibrosis stage. Our results suggest that rs738409 may have an important role in the progression of NAFLD in Japanese individuals. NAFLD is closely related with metabolic syndrome. In agreement, patients from the NAFLD group had more metabolic disorders than patients from the control group. Therefore, it is possible that the SNP dosage does not directly affect the accumulation of liver fat and/or progression of steatosis to NASH and fibrosis. We should not exclude the possibility that metabolic disorders, such as visceral adiposity and higher blood lipids, directly affect the development and progression of NAFLD.

Association of rs738409 with ALT and AST levels is controversial. In Hispanics [17], Argentinians with NAFLD [21], Italian adults with NAFLD [25], obese Italian adults [24], and obese Italian children [26], the G-allele of rs738409 was found to be significantly related to increased ALT levels. In Finnish individuals [20], obese Italian adults [24], obese Italian children [26], and Argentinians with NAFLD [21], the G-allele of rs738409 is significantly associated with increased AST levels. However, in the African American, European American [17], and German [23] populations, rs738409 is not associated with either ALT or AST levels. In our study, associations between the G-allele of rs738409 and increased levels of ALT and AST were observed only in the NAFLD group. This may be because of population bias, i.e., whether the subjects included adults or children, general population, or patients with disease (NAFLD or obesity). Variation of rs738409 was associated with NAFLD. Therefore, the association of ALT and/or AST levels with this variation may be affected by the prevalence and severity of NAFLD in the subjects of various studies. It is also likely to differ among various ethnic groups because the G-allele frequency of rs738409 in the Japanese control (44%) was similar to that in the Hispanics (49%) but higher than that in the Caucasians (23%), African Americans (14%), and Argentinians (33%).

Kollerits *et al*. [33] reported that the G-allele of rs738409 was associated with low total cholesterol and low-density lipoprotein (LDL) cholesterol but not with HDL cholesterol or triglycerides. Speliotes *et al*. [34] indicated that the G-allele is associated with decreased triglycerides levels in NAFLD patients and that this association is not observed in control subjects. In our sample population, the G-allele of rs738409 was associated with decreased levels of triglycerides in patients with NAFLD but not in control subjects. This association was significant after parameters of age, gender, BMI and treatment of dyslipidemia were adjusted (*P *= 0.00040), excluding the possibility of the effect of the treatment for dyslipidemia. SNP rs738409 was associated with liver fibrosis in our and other studies [25,34,35]. The decreased levels of triglycerides in the NALFD patients harboring the G-allele may be related to the decreased production of triglycerides due to the severity of the NASH fibrosis. The control group had no risk for NAFLD and had normal liver function. Therefore, rs738409 may not affect the triglycerides levels in subjects without NAFLD.

It has been hypothesized that NASH is induced in 2 consecutive steps (the so-called 2-hit hypothesis): (i) excess fat accumulation in the liver and (ii) subsequent necroinflammation in the liver [36]. The histological steatosis grade was higher in patients with NASH than in those with simple steatosis. Moreover, the association study showed that the risk allele (G) frequency of rs738409 was significantly higher in the NASH group than in the simple steatosis group. However, the steatosis grade was not found to be associated with the rs738409 genotype, although rs738409 has been previously associated with liver fat content [17,20,22,23]. The statistical power for association of rs738409 with the histological steatosis grade may be lower than the association with liver fat content, since the histological steatosis grade determined by the percentage of hepatocytes containing fat droplets does not represent the actual fat content. Indeed, the association between rs738409 and histological steatosis grade is controversial [25,34,35]. rs738409 is a missense variation (I148M) in *PNPLA3*. Triglyceride lipase activity of mutant PNPLA3 (I148M) was completely abolished; this led to increased triglyceride accumulation in the human hepatoma cells, which exhibited more and larger lipid droplets [37]. Furthermore, acute overexpression of the mutant protein in mice led to a 2-fold increase in hepatic fat. These experiments and several reports indicating the association between rs738409 and liver fat accumulation suggest that the I148 M variant may affect lipid accumulation in the fatty liver. Further investigations would be necessary to elucidate whether rs738409 is associated with liver fat accumulation in the Japanese population.

Serum ferritin, hyaluronic acid, and type IV collagen 7S levels were higher in the NASH patient group than in the simple steatosis group as previously reported (Table 2) [38-40]. High rates of hyperferritinemia and increased hepatic iron stores have been demonstrated in NASH patients [40]. Iron is considered a putative element that interacts with oxygen radicals in liver damage and fibrosis. There is strong evidence from in vitro and in vivo studies that iron overload enhances oxidative stress [41]. Iron can also promote fibrosis through hepatocellular necrosis and inflammation with activation of Kupffer cells (liver macrophages), which release profibrogenic mediators [42]. We showed that serum ferritin was increased in NAFLD patients harboring the G-allele. High serum ferritin levels found in hepatic iron accumulation and overload conditions enhance oxidative stress and insulin resistance. Our results and the data previous reported [25,34,35] indicate that rs738409 is likely to be related to liver fibrosis in NAFLD. Although more studies are required to elucidate the precise mechanisms, we suggest that PNPLA3 induces fatty liver and leads to necroinflammation through oxidative stress induced by high levels of iron in the liver.

Serum hyaluronic acid levels are elevated during accelerated deposition of extracellular matrix due to the upregulation of hyaluronic acid production by matrix-producing cells such as hepatic stellate cells and fibroblasts, and to the downregulation of its clearance by sinusoidal endothelial cells [43,44]. Serum hyaluronic acid appears to be a relatively accurate predictor of advanced fibrosis in NAFLD [39]. Type IV collagen is one of the extracellular matrix components produced by hepatic fibroblasts, hepatic stellate cells, and myofibroblasts. Serum type IV collagen 7S domain levels increase as fibrosis worsens as a result of synthesis by matrix-producing cells induced by liver fibrosis [45]. To ascertain whether the serum levels of hyaluronic acid and type IV collagen 7S domain are associated with rs738409, it would be necessary to examine a bigger sample of NAFLD patients, since serum levels can be affected by various conditions such as medical treatment and life-style.

## Conclusions

In conclusion, we have shown that the G-allele of rs738409 renders the Japanese population susceptible to NAFLD. We have also shown that rs738409 is associated with plasma ALT, AST, and triglycerides levels, and the fibrosis stage. Our study suggests that *PNPLA3 *may be involved in the progression of NAFLD in the Japanese population.

## Competing interests

The authors declare that they have no competing interests.

## Authors' contributions

KH performed the literature review, obtained the clinical data, and drafted the manuscript, with contributions from MY, TU, KC, AN, KN, and AS. MY, HH, HO, SM, TU, KC, and AN organized the field survey for data collection and obtained the clinical data. KH, TU, KC, AN, KN and AS were responsible for the design of the study. All authors read and approved the final manuscript.

## Pre-publication history

The pre-publication history for this paper can be accessed here:

http://www.biomedcentral.com/1471-2350/11/172/prepub

## Supplementary Material

Additional file 1 **Table S1 - Genotype frequencies and association tests of rs738409 in *PNPLA3 *in patients with NAFLD and control subjects**. The results of case-control association study performed separately in men and women. Click here for file

Additional file 2 **Table S2 - Comparison of various quantitative phenotypes among the different genotypes at rs738409 in *PNPLA3 *in patients with NAFLD and control subjects**. Mean, SD, 25^th^, 50^th^, and 75^th ^percentile of age, BMI, fasting blood sugar, HbA1c, total cholesterol, triglycerides, HDL cholesterol, systolic blood pressure, diastolic blood pressure, AST, ALT, iron, ferritin, hyaluronic acid, type IV collagen 7S, steatosis grade, and fibrosis stage among the different genotypes at rs738409 in *PNPLA3 *in patients with NAFLD and control subjects. Click here for file
