# Supplementary material for: Association of the rs738409 polymorphism in PNPLA3 with liver damage and the development of nonalcoholic fatty liver disease
Source: BMC Med Genet. 2010 Dec 22;11:172. doi: 10.1186/1471-2350-11-172 (PMC3018434; doi:10.1186/1471-2350-11-172)
Supplement: Additional file 1 — Table S1 - Genotype frequencies and association tests of rs738409 in PNPLA3 in patients with NAFLD and control subjects. The results of case-control association study performed separately in men and women. [file 1471-2350-11-172-S1.DOC]

Table S1 - Genotype frequencies and association tests of rs738409 in *PNPLA3* in patients with NAFLD and control subjects

|  | | Genotype | Risk allele (G) | *P* value (additive model) | | | OR (95% CI) | | | HWE |
| --- | --- | --- | --- | --- | --- | --- | --- | --- | --- | --- |
|  | | CC/CG/GG | Frequency | Unadjusted | Adjusted for age | Adjusted for age and BMI | Heterogyotes | Homogyotes | Additive ^*^ | *P* value |
| Men | | |  |  |  |  |  |  |  |  |
| NAFLD | | 20/59/43 | 0.59 | 0.00017 | 0.00027 | 0.0097 | 1.97 (1.07 - 3.61) | 3.56 (1.81 – 7.01) | 2.15 (1.20 - 3.83) | 0.97 |
| Control | | 58/87/35 | 0.44 |  |  |  |  |  |  | 0.82 |
| Women | | |  |  |  |  |  |  |  |  |
| NAFLD | 25/52/54 | | 0.61 | 1.6 × 10^-6^ | 0.00037 | 0.033 | 1.16 (0.69 - 1.97) | 3.66 (2.09 - 6.41) | 1.55(1.04-2.31) | 0.059 |
| Control | 117/209/69 | | 0.44 |  |  |  |  |  |  | 0.14 |

CI, confidence interval; NAFLD, nonalcoholic fatty liver disease; HWE, Hardy–Weinberg equilibrium; OR, odds ratio. ^*^ The OR was adjusted simultaneously for age and BMI using the additive model.
