# Supplementary material for: Association of the rs738409 polymorphism in PNPLA3 with liver damage and the development of nonalcoholic fatty liver disease
Source: BMC Med Genet. 2010 Dec 22;11:172. doi: 10.1186/1471-2350-11-172 (PMC3018434; doi:10.1186/1471-2350-11-172)
Supplement: Additional file 2 — Table S2 - Comparison of various quantitative phenotypes among the different genotypes at rs738409 in PNPLA3 in patients with NAFLD and control subjects. Mean, SD, 25th, 50th, and 75th percentile of age, BMI, fasting blood sugar, HbA1c, total cholesterol, triglycerides, HDL cholesterol, systolic blood pressure, diastolic blood pressure, AST, ALT, iron, ferritin, hyaluronic acid, type IV collagen 7S, steatosis grade, and fibrosis stage among the different genotypes at rs738409 in PNPLA3 in patients with NAFLD and control subjects. [file 1471-2350-11-172-S2.DOC]

Table S2 - Comparison of various quantitative phenotypes among the different genotypes at rs738409 in *PNPLA3* in patients with NAFLD and control subjects

| Quantitative |  | NAFLD ^*^ |  |  |  | Control ^*^ |  |  |
| --- | --- | --- | --- | --- | --- | --- | --- | --- |
| phenotype | CC (n=45) | CG (n=111) | GG (n=97) | *P* value ^§^ | CC (n=175) | CG (n=296) | GG (n=104) | *P* value ^§^ |
| Age (year) | 53.4 ± 11.2  (45.0; 54.0; 62.0) | 48.6 ± 15.0  (37.0; 48.0; 60.5) | 54.5 ± 16.0  (39.5; 55.5; 68.3) | 0.019 | 45.6 ± 14.9  (32.0; 46.0; 56.0) | 47.6 ± 14.5  (34.0; 49.0; 58.0) | 48.9 ± 15.7  (39.5; 50.0; 60.0) | 0.17 |
| BMI (kg/m^2^) | 26.8 ± 4.9  (23.3; 25.8; 29.4) | 28.0 ± 4.4  (24.7; 27.9; 30.1) | 27.8 ± 6.3  (24.0; 27.0; 29.6) | 0.16 | 21.2 ± 2.2  (19.6; 21.3; 23.1) | 21.4 ± 2.1  (19.9; 21.4; 23.0) | 21.4 ± 2.0  (19.9; 21.2; 23.1) | 0.75 |
| FBS (mg/dL) | 121.2 ± 28.7  (105.0; 114.0; 125.0) | 117.5 ± 38.2  (100.0; 106.0; 124.0) | 114.5 ± 33.1  (95.8; 103.5; 117.0) | 0.014 | 90.0 ± 7.8  (85.0; 90.0; 95.0) | 91.4 ± 7.6  (86.0; 91.0; 96.0) | 90.7 ± 7.4  (85.0; 91.0; 96.0) | 0.23 |
| HbA1c (%) | 6.0 ± 1.2  (5.2; 5.7; 6.7) | 5.9 ± 1.3  (5.1; 5.5; 6.3) | 5.8 ± 1.1  (5.0; 5.5; 6.1) | 0.36 | 5.0 ± 0.3  (4.8; 5.1; 5.3) | 5.0 ± 0.4  (4.8; 5.0; 5.3) | 5.1 ± 0.3  (4.8; 5.0; 5.3) | 0.78 |
| Total cholesterol (mg/dL) | 217.4 ± 32.8  (192.3; 220.5; 239.5) | 209.7 ± 40.6  (178.0; 210.0; 236.1) | 212.6 ± 37.2  (188.5; 210.0; 236.5) | 0.38 | 198.6 ± 34.0  (173.0; 198.0; 219.5) | 203.7 ± 33.7  (179.0; 204.0; 226.3) | 203.6 ± 36.6  (176.8; 202.0; 223.0) | 0.25 |
| Triglycerides (mg/dL) | 175.3 ± 81.4  (117.0; 166.0; 205.0) | 188.0 ± 119.5  (124.0; 151.5; 219.0) | 146.2 ± 69.0  (94.0; 125.0; 187.5) | 0.0055 | 75.7 ± 29.1  (52.0; 74.0; 95.0) | 76.5 ± 29.0  (53.0; 70.5; 95.0) | 72.9 ± 27.0  (54.0; 66.0; 87.0) | 0.56 |
| HDL cholesterol (mg/dL) | 54.6 ± 21.0  (43.3; 52.0; 59.8) | 50.6 ± 17.6  (41.0; 47.0; 56.5) | 53.7 ± 15.0  (43.0; 52.0; 61.0) | 0.20 | 65.4 ± 13.3  (55.0; 64.3; 74.0) | 68.0 ± 13.9  (59.0; 68.0; 76.9) | 69.1 ± 14.9  (58.0; 67.8; 77.6) | 0.073 |
| SBP (mm Hg) | 128.3 ± 14.8  (114.0; 128.0; 140.0) | 127.0 ± 16.0  (116.0; 124.0; 139.5) | 125.6 ± 15.5  (114.0; 124.0; 136.0) | 0.61 | 109.6 ± 10.6  (102.0; 110.0; 118.0) | 112.1 ± 10.0  (105.0; 113.0; 120.0) | 110.3 ± 9.7  (103.8; 110.0; 120.0) | 0.033 |
| DBP (mm Hg) | 78.9 ± 11.4  (70.0; 78.0; 86.0) | 77.1 ± 12.4  (70.0; 77.0; 88.0) | 77.3 ± 10.2  (70.5; 77.5; 83.8) | 0.88 | 68.6 ± 7.9  (62.5; 68.0; 74.0) | 70.0 ± 7.4  (65.0; 70.0; 76.0) | 69.2 ± 7.2  (64.8; 68.0; 75.0) | 0.16 |
| AST (IU/L) | 34.0 ± 14.6  (21.3; 30.0; 38.5) | 47.5 ± 26.4  (27.8; 37.0; 61.0) | 57.5 ± 35.5  (31.0; 45.0; 75.0) | 0.00017 | 19.3 ± 6.5  (15.0; 18.0; 22.0) | 19.6 ± 6.5  (15.0; 18.0; 22.0) | 21.2 ± 5.8  (17.0; 20.0; 25.0) | 0.047 |
| ALT (IU/L) | 48.3 ± 24.6  (31.0; 36.5; 61.0) | 76.9 ± 48.9  (42.8; 63.5; 94.5) | 89.6 ± 57.0  (44.8; 76.5; 116.5) | 4.7 × 10^-5^ | 15.1 ± 5.5  (10.3; 15.0; 19.0) | 15.5 ± 6.9  (11.0; 14.0; 18.0) | 16.4 ± 6.2  (11.0; 15.0; 20.0) | 0.36 |
| Iron (ng/mL) | 112.4 ± 28.7  (88.0; 123.0; 126.0) | 106.1 ± 34.7  (88.8; 107.0; 130.8) | 113.9 ± 33.1  (89.5; 109.0; 140.5) | 0.66 | ― | ― | ― | ― |
| Ferritin (ng/mL) | 168.8 ± 124.4  (82.0; 141.0; 226.2) | 271.2 ± 268.6  (83.8; 177.0; 382.3) | 276.3 ± 224.7  (148.3; 208.2; 350.4) | 0.038 | ― | ― | ― | ― |
| Hyaluronic acid  (ng/dL) | 48.4 ± 47.4  (23.0; 33.5; 46.3) | 39.7 ± 55.3  (10.0; 21.0; 42.8) | 57.3 ± 74.0  (16.5; 37.0; 86.8) | 0.022 | ― | ― | ― | ― |
| Type IV collagen 7S (ng/dL) | 4.6 ± 1.9  (3.9; 4.2; 4.9) | 4.6 ± 1.4  (3.7; 4.5; 5.2) | 4.6 ± 1.2  (3.6; 4.5; 5.2) | 0.76 | ― | ― | ― | ― |
| Steatosis grade | 1.5 ± 0.6  (0.0; 1.0; 1.0) | 1.6 ± 0.7  (0.0; 1.0; 1.0) | 1.6 ± 0.6  (1.0; 1.0; 1.8) | 0.57 | ― | ― | ― | ― |
| Fibrosis stage | 0.7 ± 0.8  (1.0; 1.0; 2.0) | 1.1 ± 0.9  (1.0; 2.0; 2.0) | 1.2 ± 1.0  (1.0; 2.0; 2.0) | 0.013 | ― | ― | ― | ― |

Data are represented as the mean ± SD (25^th^, 50^th^, and 75^th^ percentile). AST, aspartate transaminase; ALT, alanine transaminase; BMI, body mass index; DBP, diastolic blood pressure; FBS, fasting blood sugar; HbA1c, hemoglobin A1c; HDL, high-density lipoprotein; NAFLD, nonalcoholic fatty liver disease; SBP, systolic blood pressure. ^*^ The data of each quantitative phenotype from NAFLD and control subjects were compared for the different rs738409genotypes in patients with NAFLD and control subjects. ^§^ *P* values were analyzed using the Kruskal–Wallis test in each group of NAFLD and control subjects.
